# Occlusal considerations in maintaining health of implants and their restorations

**DOI:** 10.1038/s41415-024-7407-7

**Published:** 2024-05-24

**Authors:** Charlotte Stilwell

**Affiliations:** Specialist in Prosthodontic Dentistry, Specialist Dental Services, Harley Street, London, W1G 7HX, UK; Division of Gerodontics and Removable Prosthodontics, University of Geneva, Geneva, Switzerland

## Abstract

Dental implants are a regular feature in daily clinical practice and there is a need to undertake routine assessment and maintenance of implants and their restorations on par with that provided for natural teeth. Occlusal checks form an important part of the maintenance regime for preserving the integrity of implants, their restorations, and health of the peri-implant tissues. Implant restorations are subjected to the full characteristics and magnitude of occlusal forces, including those associated with parafunction. Compared with the periodontal ligament around teeth, the biophysical response to occlusal forces of osseointegration is different through the more rigid link of implant to bone and reduced proprioception. Risks attributable to occlusal forces primarily affect implant restorations and they are elevated in the presence of bruxism. The occlusal guidelines recommended by the literature are aimed at reducing these risks and regular assessment and maintenance of the occlusion is essential. A four-step sequence is presented to ensure that the annual occlusal checks include the patient's input and evaluation of restoration integrity, occlusal scheme, additional protection, and spatial changes.

## Key points

- This paper provides an overview of recommended occlusal checks as part of an annual maintenance regime for dental implants, their restorations and peri-implant tissues.
- The literature suggests that hardware complications are the main risks arising from the biophysical properties of osseointegration and consequent response to occlusal forces.
- For completeness, the occlusal checks can be approached in a four-step sequence to include the patient's input as well as evaluation of restoration integrity, occlusal scheme, additional protection, and spatial changes.

## Introduction

Dental implants are fast becoming a regular feature in daily clinical practice. Regardless of whether primary dental care practitioners are actively engaged in placing and restoring implants, there is a need to undertake routine assessment and maintenance of implants and their restorations on par with that provided for natural teeth.

Within this themed Issue on implant maintenance in general dental practice, the specific focus of this article is on the occlusal considerations and checks for maintaining the integrity of implants, their restorations, and health of the peri-implant tissues.

## Occlusal considerations for dental implants and restorations

Implant restorations are subjected to the full characteristics and magnitude of occlusal forces, including those associated with parafunction.^1^^,^^2^ The biophysical properties of osseointegration are different to the periodontal ligament, particularly in the ability to mitigate the impact of occlusal forces.^3^^,^^4^^,^^5^^,^^6^

The question is how the more rigid link between implants and bone and their reduced proprioception impacts on the risk of complications from occlusal overload^7^ and consequently, what occlusal checks need to be considered at annual visits for maintenance. The following brief overview will discuss risk of potential hardware complications (implants, restorations, and restorative components) and biological complications (osseointegration, peri-implant tissue and crestal bone levels) as well as risks posed by the given occlusal scheme.

### Risks of hardware complications

**Fig. 1:**
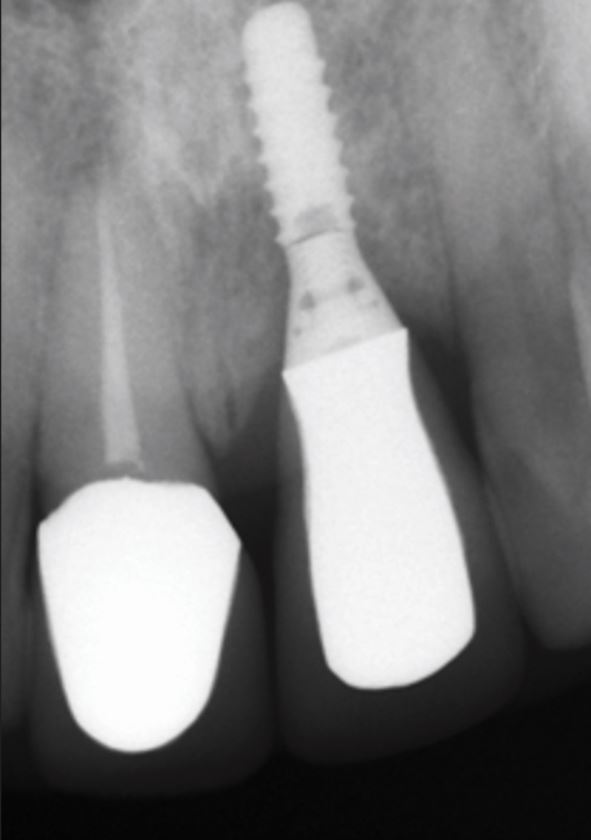
Fractured implant secondary to buccal crestal bone loss and occlusal overload

**Fig. 2:**
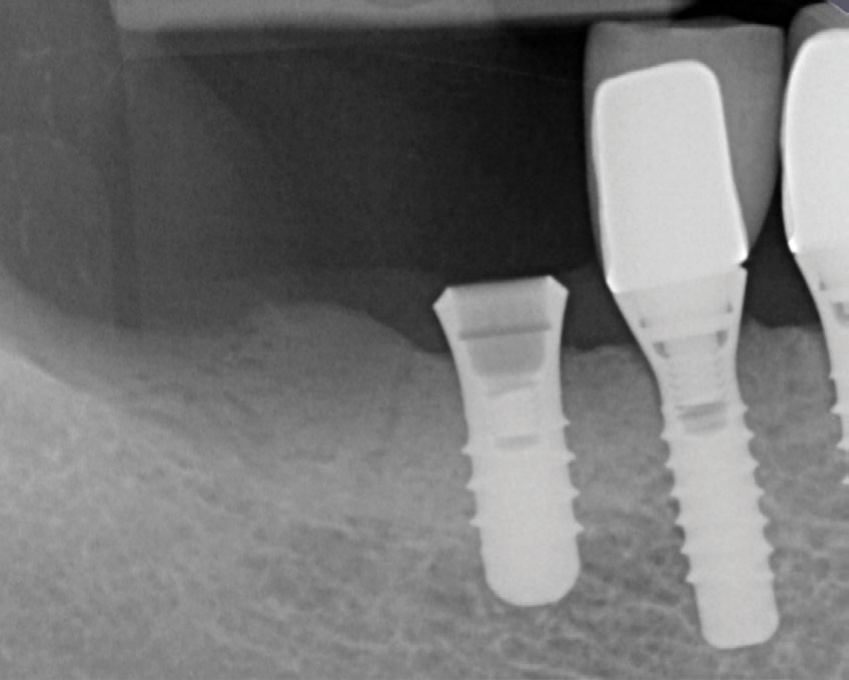
Fractured and retained abutment screw in bruxist patient

Occlusal overload can cause implant fracture (Fig. 1), but it is a rare occurrence with a reported incidence around 0.5%.^8^ The most frequent mechanical and technical complications for the implant restorations over ten years have been reported as ceramic chipping (20.31%), occlusal screw loosening (2.57%), abutment and screw loosening (5.3%) and loss of cement retention (2.06%).^9^ With the high and frequent complication of ceramic chipping alone, the direct or indirect relationship with occlusal forces is clearly an important consideration for implant restorations, particularly in the presence of bruxism^10^ (Fig. 2).

### Risks of biological complications

Occlusal overload as a risk factor for biological complications is less clear. The process of osseointegration can be compromised by mechanical loading.^11^ The effect of implant overload on bone/implant loss in clinically well-integrated implants is poorly reported and there is little unbiased evidence to support a cause-and-effect relationship,^12^^,^^13^ or that bruxism is a risk factor for biological complications.^10^

### Risks posed by given/prevailing occlusal scheme

General risk assessment for implant therapy should include assessment of occlusal complexity and identification of occlusal risk factors. In the SAC Classification for implant dentistry,^14^ occlusal risk is elevated in the absence of anterior teeth guidance within a prevailing occlusal scheme, with direct involvement of implant restorations in guidance and in the presence of parafunction.

## Loss of interproximal contact between implant restorations and adjacent teeth

**Fig. 3:**
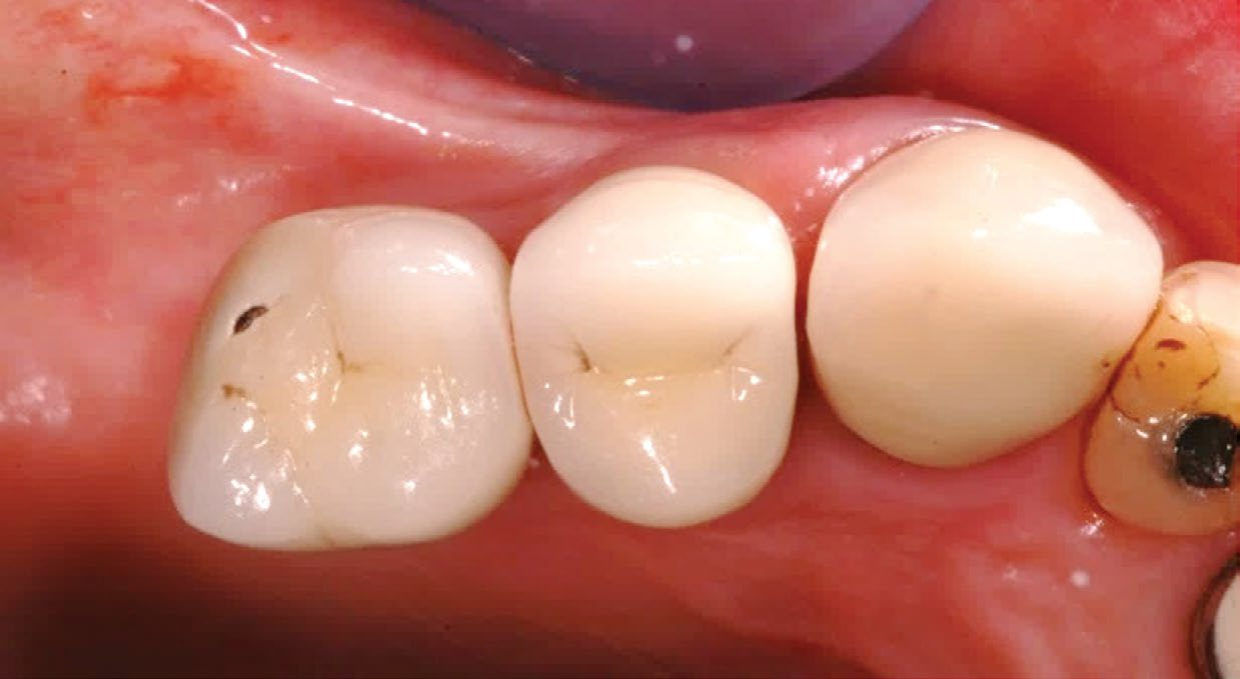
Occlusal view of implant restorations in upper right premolar sites. UR5 has ceramic cusp fracture and there is loss of the originally established interproximal contact (ICL) between 14 and natural tooth 13

Interproximal contact loss (ICL) is described as absence of a previously established interproximal contact between the implant prosthesis and adjacent teeth^15^(Fig. 3). ICL is reported as a frequent prosthodontic complication affecting approximately 29% of contact points, predominantly on the mesial aspect of implant restorations and becoming more common the longer restorations have been in function.^16^^,^^17^

The clinical concern centres on potential food impaction, damage to the interproximal tissues, potentially difficult and expensive interventions to reestablish proximal contact, and a continued risk of reoccurrence. Definitive causation of ICL is not established but hypotheses include mesial drift of the remaining teeth and morphological changes related to continuous facial growth.

The literature does not suggest a direct causative link to occlusal forces, but ICL represents an occlusal change which justifies mention of the condition here.

## Occlusal checks as part of annual maintenance regime

Occlusal checks can be divided into four steps. The first step is to obtain occlusally relevant information from the patient. The second step examines the implant restoration and its integrity. The third step assesses the occlusal scheme and any additional protection. The fourth step evaluates the spatial relationship of the implant restoration to adjacent teeth/restorations.

### Step 1: patient input

The patient is an important source of information with occlusal relevance volunteered or obtained through questioning. For a start, it is relevant to know whether any occlusally related treatment has been undertaken since the last check-up. Other concerns and information are seen in Table 1.

**Table 1:** Patient information and concerns with direct or indirect occlusal relevance

Step 1: Occlusal history as part of annual maintenance regime	
Patient input/concerns	**Occlusal event since last visit** Restoration refitted/repaired/updated elsewhere since last check-up
	**Damage or change, visible in smile or perceived** Chipping or fracture Spatial difference in length or appearance of gap
	**Discomfort/symptoms** Teeth/mucosa Masticatory Food retention Temporomandibular
	**Elevated awareness of bite** Conscious of change in bite, general/localised, heavy or loss of contact Food packing
	**Retention** Movement perceived/possible loosening Loss of restoration/overdenture attachment

**Fig. 4:**
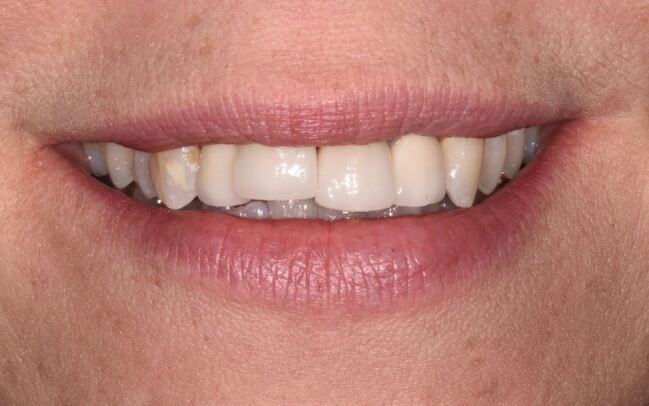
Continued alveolar growth in young woman has resulted in incisal level discrepancy between 11 implant crown and 21 natural tooth

Occlusally related concerns could be damage to edges of implant restorations such as ceramic chipping and discolouration. It could also be a change in the spatial relationship of an implant restoration and the adjacent teeth related to continued alveolar growth^18^^,^^19^^,^^20^ (Fig. 4) or an interproximal gap that is visible in the smile as a result of occlusal instability/overload or marginal bone loss of adjacent teeth.^21^

The patient could mention symptoms from adjacent or opposing teeth or from edentulous ridges under implant overdentures. There could be mention of food packing related to ICL^22^ or a restoration fracture involving an interproximal contact. Patients with implant dentures could comment on increasing difficulties with chewing food due to wear of the denture teeth.

Reported discomfort and symptoms could point to bruxism^23^ and temporomandibular dysfunction (TMD).^24^^,^^25^ Concerns could also be related to perceived changes in the bite. These could be more general or localised and include raised awareness of a heavy contact on a front tooth implant, difference in ability to incise and chew food due to loss of contact or grating sounds indicative of dynamic interferences.

**Fig. 5:**
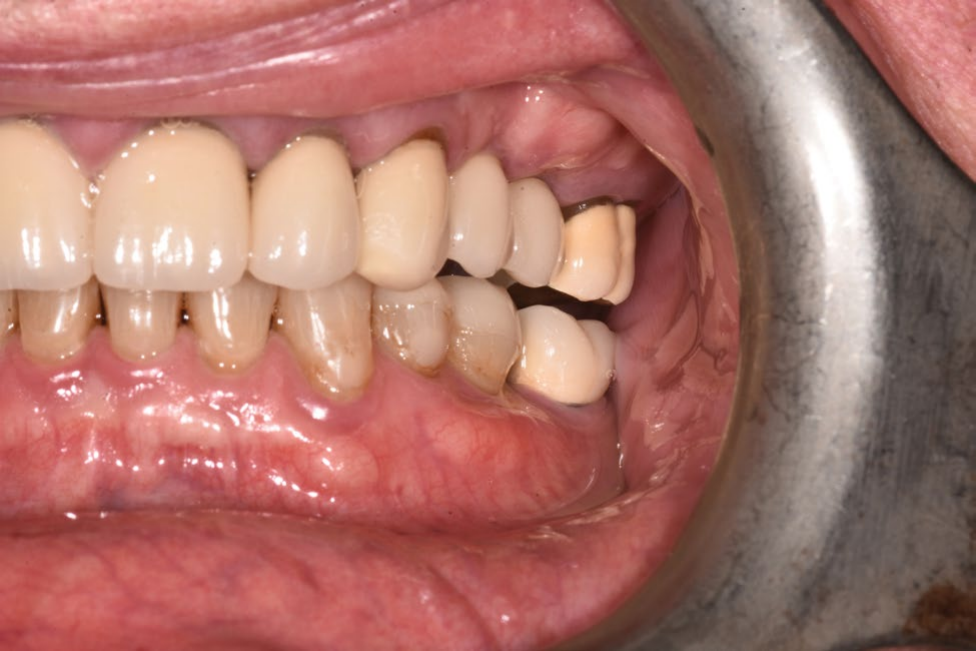
Continued changes to tooth positions and mandibular postural change has resulted in combination of infra-occlusion of implant restorations in sites 24, 25 and 36 as well as left side posterior open occlusion in centric closure

The patient may report a more extensive change to the bite that could suggest a change in mandibular posture (Fig. 5). Here, signs of an acute or more chronic TMJ or muscle disorder should be investigated, and onward referral considered.^26^^,^^27^

With retention issues, patients are often able to detect screw loosening early and report a slight clicking sensation during tooth contact or eating. With cementation failure, a single restoration is likely to come away with risk of loss. For multi-unit restorations, the patient may report movement or increased awareness. Report of loss of retention or retentive components in overdentures is also to be expected.^28^

### Step 2: restoration integrity

The occlusal checks of the restoration in Table 2 start with checking the retention of the restoration. There should be no detectable movement in screw-retained restorations and the cement-retained restoration should resist light finger pull. Early signs of loosening for either type of restoration can be difficult to detect so look for gap or fluid movement at the restoration margin and movement of the restoration in dynamic occlusion.

**Table 2:** Occlusal evaluation of implant restorations

Step 2: Occlusal evaluation of implant restorations		
Restoration integrity	Retention	**Mobility detected for screw-retained:** Identify loosened screw (abutment and/or restoration), consider replacing screw(s) and retorque to value(s) recommended by implant manufacturer for the relevant component(s) **Loss for cement-retained:** Clear all existing cement from abutment and restoration and refit using minimal amount of readily removed cement (for example, zinc phosphate)
	Surface finish	Return to smooth and high shine to reduce risk of causing attrition of opposing unit Crack lines to be kept under observation
	Chipping and fractures	Localised minor might be easily smoothed and polished Greater defect in need of repair or replacement Structural necessitating fundamental repair of replacement
	Wear	Less likely for metal/ceramic surfaces More likely for PMMA teeth used; for example, in implant overdentures As yet minor to be monitored only In need of replacement of teeth
	Access hole seal	Intact and conforming with the restoration occlusal morphology Compromised and in need of repair or replacement

**Fig. 6:**
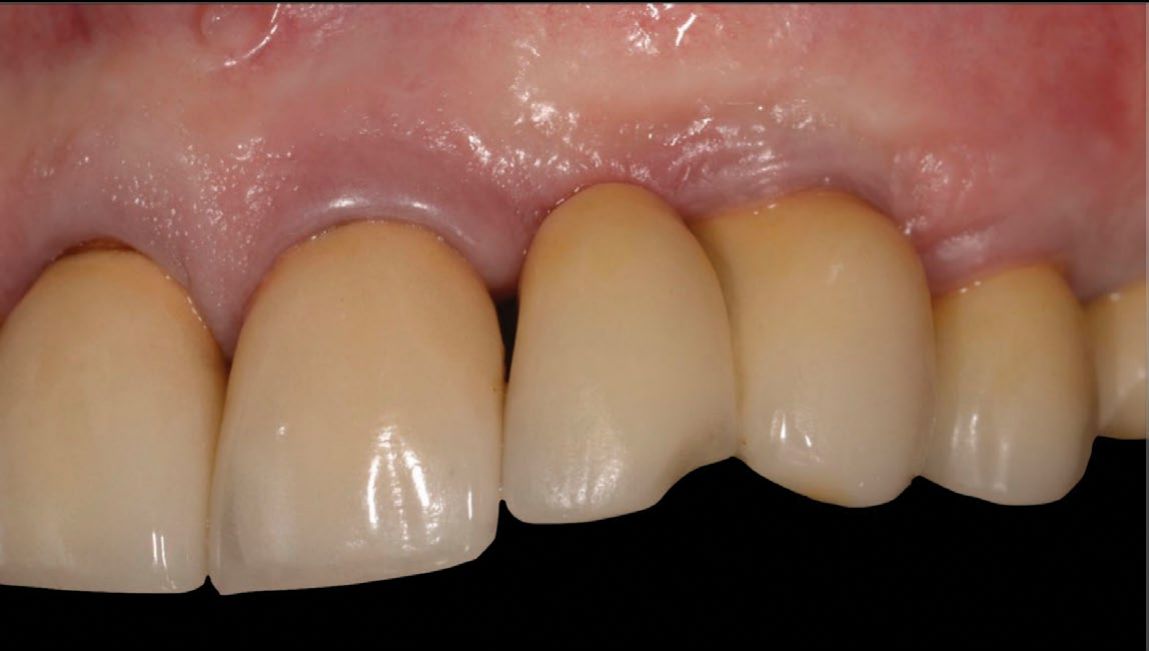
Chipping repaired by direct composite restoration. Images courtesy of Professor Irena Sailer

**Fig. 7:**
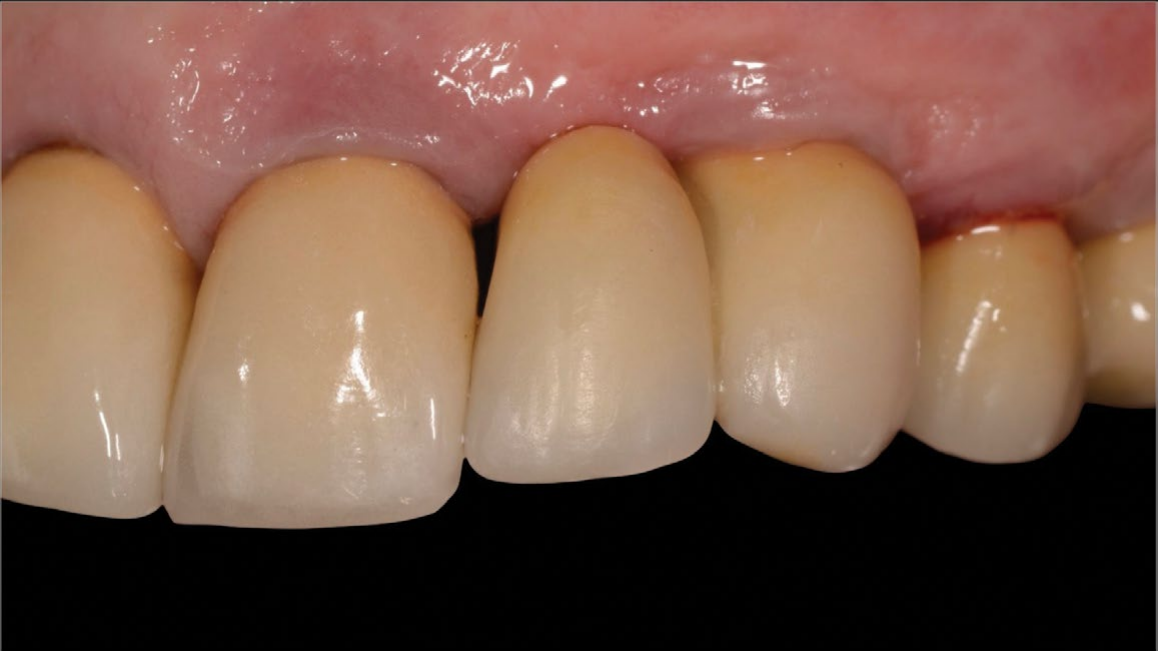
Chipping repaired by direct composite restoration. Images courtesy of Professor Irena Sailer

Next, is checking the restoration for crack lines, chipping, or structural fractures. If present, decision is required regarding intervention. Crack lines should be documented, and occlusal contacts checked. Smoothing and polishing may suffice for minor chipping. For larger ceramic chipping, such as an incisal corner, direct bonded composite or an indirect ceramic repair may be a solution^29^ (Figures 6 and 7). Structural fractures with loss of significant parts of an occlusal surface or interproximal contact are likely to require complete replacement of the restoration.

For screw-retained restorations, the degradation or loss of the closure filling in the access hole is a frequent complication. Before replacement, the screw torque recommended by the implant manufacturer should be checked using a torque device and the screw head should be protected against damage on future re-entry. Polytetrafluoroethylene (PTFE) tape is recommended for this purpose as an inert material with favourable properties.^30^ Direct composite for either longer- or shorter-term use is used depending on when anticipated access is required again.

If the entire restoration needs to be replaced, the design of the failed restoration and the dimensions of the restorative space should be assessed to identify possible weaknesses and improvements needed to reduce the risk of recurrence.^29^ Examples are review of the framework material with chipping and size and shape of connectors for multi-unit restorations.

### Step 3: occlusal scheme

The third step is to assess the current occlusal scheme against recommended occlusal guidelines. In Table 3, these guidelines^2^^,^^31^ are divided into contacts in static closure and contacts during dynamic movement.

**Table 3:** Evaluation of occlusal scheme and additional occlusal protection

Step 3: Occlusal evaluation of occlusal scheme and protection	
Occlusal scheme for protection of implant restorations	**Recommended contact design for implant restorations** **Static contacts:** Anteriors have long centric freedom (shimstock pulls through easily) Posteriors are centred in wide occlusal fossa Graded contact in mixed teeth and implants dentitions to allow for difference in resilience between periodontal ligament and osseointegration **Dynamic contacts:** Anterior guidance by natural teeth where present and possible Anterior guidance by implant restorations Limited to materially strong aspects of palatal surface Absence of simultaneous posterior interferences Absent on posterior implant restorations **Corrections needed with:** Anterior premature static and dynamic contacts Posterior premature static contacts and dynamic contact interferences Polish to ensure smoothness and high shine of the restoration surface
Additional occlusal protection	**Not needed or already present** No signs or symptoms of active bruxism or damage to restorations Existing hard appliance conforming to prescribed scheme (usually mutual protection) or satisfactory Essix type guard **Occlusal guard/splint indicated** Active bruxism and implant/restoration complications induced by occlusal force Hard appliance with mutually protected scheme or Essix type retainer(s)

#### Static occlusal scheme

In static closure, occlusal contacts should be in occlusal fossae and on the cingulum for posterior and anterior implant restorations, respectively. They should also be on dimensionally adequate and well-supported aspects of the restoration material. If contact can be placed on the access hole filling in screw-retained restorations, it is easy to adjust and renew as required.

#### Graded contact between lighter contact and firm closure

With a mix of teeth and implant-supported restorations, graded contact is used to address the difference in the vertical, physiologic tooth mobility in the periodontal ligament between first light contact and full firm closure versus virtual absence of the same in the osseointegration between implants and bone.^32^ The intention is for implant restorations to only come into full contact in firm closure of the teeth.

**Fig. 8:**
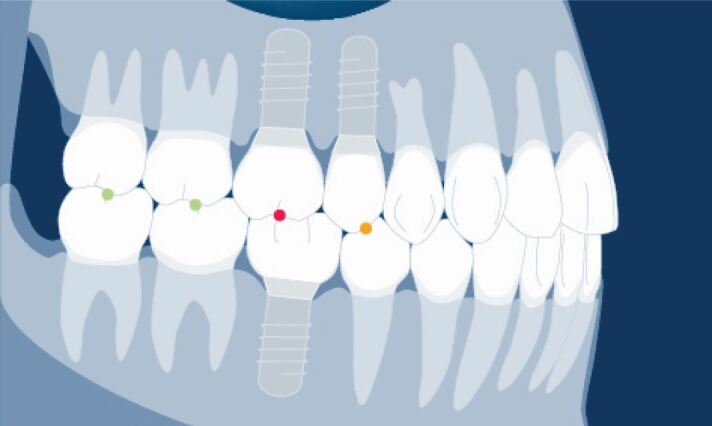
Illustration of graded contact in first light contact of the natural teeth. Shimstock holds between the molar teeth with the green dots, just pulls through between the upper premolar crown and lower premolar tooth and implant with the yellow dot, and pulls through easily between the opposing implant molar crowns with the red dot. Graphic used with permission of the ITI International Team for Implantology

**Fig. 9:**
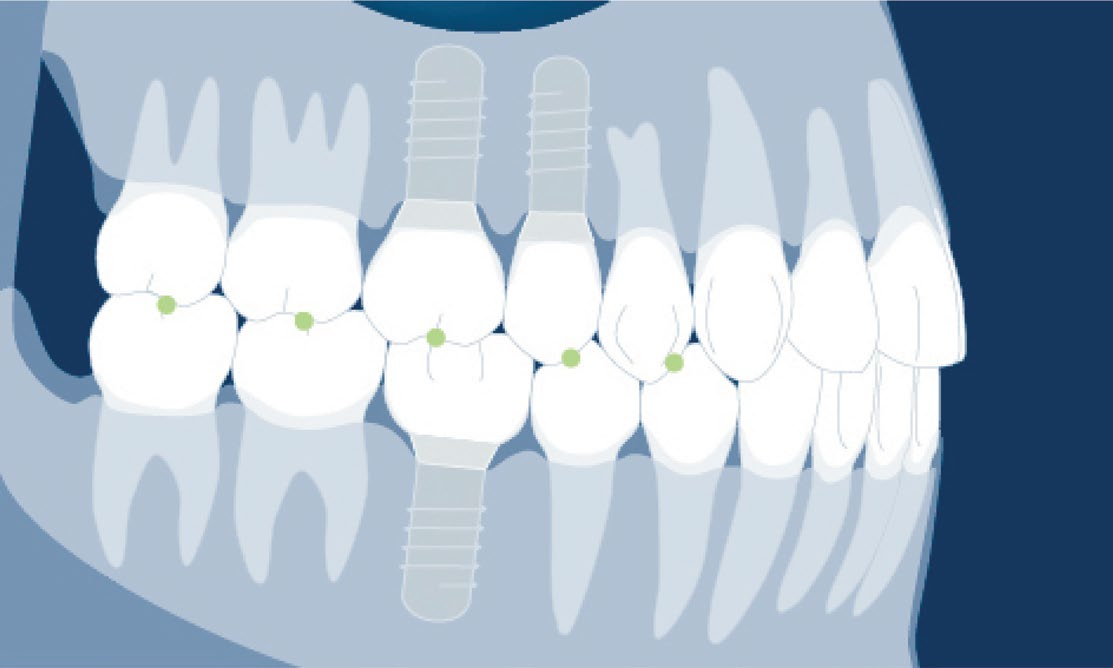
Illustration of graded contact in firm closure of all teeth and implant restoration where all hold Shimstock as depicted by the green dots. Graphic used with permission of the ITI International Team for Implantology

This is illustrated in Fig. 8 where only the opposing natural teeth should hold the Shimstock foil in light contact. Where an implant restoration opposes a natural tooth, it should just pull through and where two implant restorations are opposing one another, it should pull through easily. In firm closure, all opposing posterior teeth and implant restorations should hold Shimstock foil (Fig. 9).

For anterior implant restorations,^31^ a long centric freedom is recommended even in firm closure, that is, Shimstock just pulls through against an opposing anterior tooth and pulls through easily against an opposing implant restoration.

#### Dynamic occlusal scheme

Where present and possible, natural teeth should provide anterior guidance to take advantage of their biophysical properties, particularly in the initial guidance. Where implant restorations provide part or all of the anterior guidance, there should be no simultaneous posterior dynamic interferences.

For posterior implant restorations, loading should be limited to static contact and therefore no dynamic contacts. The occlusal fossa should be wide, and the cusps‘ angles shallow if not conflicting with aesthetic requirements.

#### Occlusal corrections as part of maintenance

Occlusal adjustments are frequently required as part of continuing maintenance.^28^ This applies to both static and dynamic contacts to relocate contacts, reinstate graded contact and eliminate interferences. On completion, the affected surfaces should be repolished to a high shine/lustre.

#### Protection for night-time use against parafunctional occlusal forces related to bruxism

The occlusal check should also look for signs of active parafunction. Scalloping of the lateral borders of the tongue and a hyper-keratinised crest in the cheeks should be explored for relationship with habit or bruxism.^33^

**Fig. 10:**
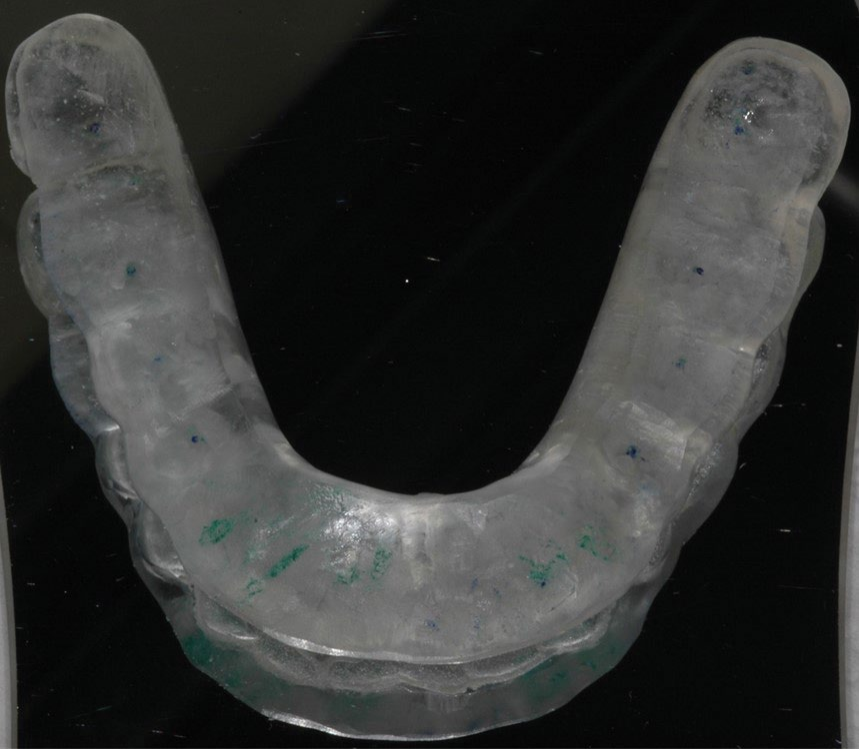
Mandibular hard occlusal appliance with mutually protected occlusal scheme for night-time protection against bruxism-induced occlusal forces

In the presence of bruxism, occlusal checks of implant restorations are even more important and consideration of a hard occlusal splint prudent. In addition to protection, they are also a useful if nonspecific clinical instrument for detecting wear facets and diagnosing active bruxism (Fig. 10).

Existing protection should be checked to confirm that it is in satisfactory condition with an effective occlusal scheme, usually mutual protection.^31^

### Step 4: restoration relationship to adjacent teeth/prostheses

Table 4 sets out checks for ICL and for corono-apical position changes. For the management of ICL, two factors should be considered before intervention. The first is the high possibility of reoccurrence and the second is that correction could be costly and difficult.

**Table 4:** Occlusal evaluation of spatial changes between implants restorations and adjacent teeth

Step 4: Occlusal evaluation of spatial changes between implants restorations and adjacent teeth		
Restoration relation to adjacent teeth/prostheses	Proximal contacts	**Present or as originally established at point of fitting** **Absent but stable condition:** Can be monitored (no caries, peri-implant inflammation, crestal bone loss) **Unstable condition in need of correction:** Addition to contact point adjacent tooth or on restoration Replacement of restoration
	Vertical relation	**Change detected** With no aesthetic or functional concern Monitor With aesthetic and or function concern in need of correction: Modification of existing restoration (for example by addition of adhesive veneer/onlay) Replacement of restoration

If the patient is not raising any concerns and no caries or periodontal/peri-implant issues are detected, the most straightforward approach is to provide specific oral hygiene instruction and simply monitor the site.

Intervention is indicated if the patient is complaining of food retention or in presence of disease.^34^ The simplest way to re-establish a contact point is to add composite to the adjacent tooth. Otherwise, replacement of an existing restoration in the adjacent tooth or modification or replacement of the implant restoration may be needed. A retrievable restoration makes this simpler and is one of the advantages of screw-retained over cemented-retained.

If correction of infra-occlusion is indicated on either aesthetic and or functional grounds, use of resin-bonded onlays or veneers may be a solution.^29^ This is particularly helpful where the restoration is not easily retrieved. The alternative is complete replacement of the restoration. The patient should be aware that the correction would only address difference at the incisal/occlusal aspect and any discrepancy in the marginal soft tissues would either have to be accepted or require separate intervention.

## Conclusion

The risks attributable to occlusal forces primarily affect implant restorations. With multiple hardware complications and in the presence of active bruxism, risks are elevated. The occlusal guidelines recommended by the literature are aimed at reducing these risks and regular assessment and maintenance of the occlusion is essential. The four-step sequence presented further ensures that the annual occlusal checks include the patient's input and evaluation of restoration integrity, occlusal scheme, protection, and spatial changes.
